# Multi-Channel Fetal ECG Denoising With Deep Convolutional Neural Networks

**DOI:** 10.3389/fped.2020.00508

**Published:** 2020-08-26

**Authors:** Eleni Fotiadou, Rik Vullings

**Affiliations:** Department of Electrical Engineering, Eindhoven University of Technology, Eindhoven, Netherlands

**Keywords:** convolutional neural networks, encoder-decoder network, fetal ECG denoising, fetal ECG enhancement, fetal electrocardiography

## Abstract

Non-invasive fetal electrocardiography represents a valuable alternative continuous fetal monitoring method that has recently received considerable attention in assessing fetal health. However, the non-invasive fetal electrocardiogram (ECG) is typically severely contaminated by a considerable amount of various noise sources, rendering fetal ECG denoising a very challenging task. This work employs a deep learning approach for removing the residual noise from multi-channel fetal ECG after the maternal ECG has been suppressed. We propose a deep convolutional encoder-decoder network with symmetric skip-layer connections, learning end-to-end mappings from noise-corrupted fetal ECG signals to clean ones. Experiments on simulated data show an average signal-to-noise ratio (SNR) improvement of 9.5 dB for fetal ECG signals with input SNR ranging between −20 and 20 dB. The method is additionally evaluated on a large set of real signals, demonstrating that it can provide significant quality improvement of the noisy fetal ECG signals. We further show that employment of multi-channel signal information by the network provides superior and more reliable performance as opposed to its single-channel network counterpart. The presented method is able to preserve beat-to-beat morphological variations and does not require any prior information on the power spectra of the noise or the pulse location.

## Introduction

The fetal electrocardiogram (ECG) can be used to monitor the condition of the fetal heart from early pregnancy until delivery (1). Nowadays, fetal monitoring is mainly performed by cardiotocography or by ECG recordings where an electrode is directly placed on the fetal scalp. Cardiotocography records the fetal heart rate together with the uterine contractions. The advantages of the method are that it is performed non-invasively and is safe for the patient. On the other hand, it is prone to signal loss, while recorded changes of the heart rate are not always precise (2). Scalp ECG recordings are a more reliable means of monitoring the fetal health. However, they are invasive, may pose a health risk to the fetus, and can only be performed during labor, when the membranes have ruptured.

Non-invasive fetal electrocardiography, performed by placing electrodes on the maternal abdomen, is a promising alternative to standard fetal monitoring. In comparison with cardiotocography, it provides more accurate information because it does not need to average over multiple beats for the heart rate extraction. Moreover, it provides the possibility to assess the ECG morphology, related to the electrical activity of the fetal heart. The advantage of the method over the scalp ECG measurements is that it can be performed already during pregnancy, it is safe for the fetus and comfortable for the mother. However, the difficulty to extract a clean fetal ECG from the abdominal mixture is the main reason that the application of the method in clinical practice is still limited. The interferences and noises in the abdominal recordings among others include the maternal ECG, powerline interference, baseline wander, muscle noise from the fetus and mother and movement artifacts. Considering that the signals of some of these interferences overlap both in time and frequency with the fetal ECG, the extracted fetal ECG signals usually have very low signal-to-noise ratio (SNR). Therefore, the non-invasive recordings are in practice merely used for heart rate analysis.

There are typically three main steps in the fetal ECG extraction process; preprocessing, separation and postprocessing (3). Preprocessing includes removal of unwanted noise such as powerline interference and baseline wander. In the separation step, the maternal ECG is estimated and then subtracted from the signals to obtain the fetal ECG. Finally postprocessing is employed to enhance the quality of the extracted fetal ECG signals. The work on non-invasive fetal ECG analysis has mainly targeted the first two steps, together with the improvement of the acquisition devices (4), while only few works focused on the postprocessing of the obtained signals. Beat-to-beat averaging is a traditional method which is often used to improve the SNR of the extracted signals, at the expense of losing individual variations in pulse shape (5). Different wavelet denoising techniques were additionally proposed in the literature for the postprocessing of the extracted fetal ECG signals (6, 7). In a previous work (8), the authors employed an augmented time-sequenced adaptive filter to enhance the quality of the extracted fetal ECG. Despite the significant quality improvement that the method achieves, the location of the fetal pulses is required to synchronize the filter and the method cannot handle abrupt changes in fetal ECG morphology, e.g., in cases of arrhythmia.

Recently, deep neural network models such as convolutional neural networks (CNNs), recurrent neural networks (RNNs) and stacked denoising autoencoders have been successfully applied for a variety of purposes including signal and image denoising (9–13). Moreover, few works reported adult ECG signal denoising (14, 15), fetal QRS detection (16, 17), and fetal ECG signal reconstruction (18). Zhong et al. (19) presented a deep convolutional encoder-decoder framework for preprocessing abdominal recordings to remove noise. However, they did not extract the fetal ECG from the preprocessed signals to ensure that it is not suppressed by the network. The authors were the first to propose a deep convolutional encoder-decoder network for postprocessing non-invasive single-channel fetal ECG (20, 21), achieving a substantial quality improvement of the noisy signals. The method tackled some of the shortcomings of the state-of-the-art non-invasive fetal ECG postprocessing methods, since it can preserve beat-to-beat morphological variations and does not require prior knowledge about the location of the fetal pulses. However, in cases of heavily corrupted signals, the method was unable to reliably reconstruct some relevant morphological features of the ECG, sporadically even causing presence of “fake” waves, i.e., waves in the reconstructed ECG that should not have been there or should have had opposite sign. For a practical application this might be dangerous, leading to wrong diagnosis.

In this work, we are dealing with the aforementioned problem by extending our model to handle multiple fetal ECG channels. Multiple electrodes measure the electrical activity of the heart from different angles. We propose to use a deep convolutional encoder-decoder network with symmetric skip connections that learns how to optimally combine the input channels to deliver a reliable clean, multi-channel ECG as output. The method eliminates the residual noise in the fetal ECG by capturing the signal structure in the convolutional layers and recovering the details by the transposed convolutional layers.

## Materials and Methods

### Data

#### Simulated Data

For the training, but also for the evaluation of the proposed network, we created an extensive simulated fetal ECG dataset that consists of two parts. The first part was built by employing the fecgsyn toolbox developed by Behar et al. (22, 23). The toolbox enables the creation of abdominal mixtures with adjustable noise sources, heart rate, heart rate variability, fetal movement, ectopic beats and contractions. A Gaussian model is used to simulate the ECG beats, as originally developed by McSharry (24) and further improved by Sameni (25). Any number of electrodes can be positioned on the maternal abdomen for the simulations. Unfortunately, the simulated fetal ECGs are based merely on 9 available vectorcardiograms (VCGs). Since there is limited variation in the shape and lengths of the individual PQRST waves in these VCGs, there is an increased risk of overfitting the network. This means that the network might learn to reproduce these limited morphologies and enforce resemblance of the denoised signals with the training data. In fact, what happened in our initial experiments is that the P and T waves of the denoised signals were shifted with respect to their ground truth data to match the locations of the training data. For this reason, we built a modified version of the toolbox that creates a variety of new ECG morphologies based on the already available VCGs. The modified toolbox receives a VCG as input, alters the length of the VCG intervals along with the amplitudes of the PQRST waves and subsequently uses it as a base to form the abdominal fetal ECG. Initially, for all 9 VCGs, the points of interest, which are the beginning and end of the P wave, T wave, and QRS complex were annotated and saved to be later available to the simulator. In every iteration of the modified simulator, one of the 9 VCGs is randomly selected and subsequently the start and the end of the waves are randomly shifted in position. Since the shift of the start and shift of the end point of each wave are not identical, also the length of the waves is automatically varied this way. The amplitude of each wave is changed as well by random scaling. The modified VCG is the starting point that the abdominal fetal ECG can be created. With the help of the modified toolbox we created a large dataset of four-channel abdominal mixtures, where different physiological events were considered, such as heart rate decelerations and accelerations, fetal movement, ectopic beats, uterine contraction etc., similar to the Fetal ECG Synthetic Database (26). The VCG alterations were chosen so as to include an ample range of variations of the ECG morphological features, while still ensuring their physiological plausibility. When obtaining the simulated data, we varied the placement of the four electrodes to make the method invariant to variations in the electrode position.

To further enrich the ECG morphologies in our dataset and reduce the risk of overfitting to the training data we generated an additional set of simulated signals based on adult ECG from the PTB Diagnostic ECG Database of Physionet (27). The database comprises of both normal and pathological signals with 15 leads, sampled at 1,000 Hz. 549 records from 290 male and female subjects are available. Adult and fetal ECG have similar morphology but the adult ECG intervals and amplitudes are larger compared to the fetus. The adult ECG was preprocessed to remove noise and resemble the fetal ECG. First, a high-pass filter with cut-off frequency of 1 Hz was applied followed by Savitzky-Golay filtering of order 8 and length 31. Afterwards, considering that the fetal heart beats two to three times faster than the adult heart, the signals were resampled to half frequency. Adjustment of the signals amplitude was not necessary because they were, in a later data preparation step, anyway normalized before entering the network. As a next step, four-channel signals were created by making random combinations of four leads, where a maximum of two was chosen out of the six first limb leads. Finally, “real” noise was added to the signals. For the “real” noise we employed a set of six-channel abdominal recordings of an ongoing study of which the protocol is described in (28). In a subset of these recordings we found it impossible to detect the fetal ECG, either because of the shielding of the fetus by the vernix caseosa or because the fetal heart was far from some electrodes. We considered that these measurements, after the maternal ECG suppression and powerline interference removal, consist of pure noise and added them to the preprocessed adult ECG to generate our simulated fetal ECG signals.

#### Real Data

In order to evaluate how well our algorithm performs in real signals we employed two databases. The first one is a private set of non-invasive fetal ECG measurements, obtained in collaboration with the Máxima Medical Center, Veldhoven, the Netherlands (28, 29). The dataset contains 462 six-channel recordings of different women, at least 18 years old, between 18 and 24 weeks of gestation. The fetal ECG was recorded with adhesive Ag/AgCI electrodes on the abdomen of the pregnant women while they were in semi-upright position. Six electrodes were placed around the navel to produce six channels of electrophysiological measurements, while two additional electrodes, placed close to the navel, served as common reference and ground. Each recording lasted from 5 up to 50 min and was digitized and stored at 500 Hz sampling frequency by a fetal monitoring system (Nemo Healthcare BV, The Netherlands). Since the signals were measured through six electrodes, we selected the first, third, fourth and fifth dimensions to form the four-channel fetal ECG signal.

The second real dataset is the Abdominal and Direct Fetal Electrocardiogram Database which consists of four-channel abdominal fetal ECG recordings obtained by five women in labor, between 38 and 41 weeks of gestation (30). Each recording comprises four different signals acquired from the maternal abdomen together with a reference direct fetal ECG registered from the fetal head. The configuration of the abdominal electrodes consisted of four electrodes placed around the navel, a reference electrode placed above the pubic symphysis and a common reference electrode placed on the left leg. The recordings have duration of 5 min and are sampled at 1,000 Hz.

#### Data Preprocessing

The signals of all the datasets were preprocessed before entering the network. The fetal ECG extraction was performed with the help of the open-source algorithm of Varanini et al. (31) and the signals were resampled to 500 Hz to have a common reference. Finally, the fetal ECG signals were divided in segments of 1920 × 4 samples and normalized to have zero mean and unity standard deviation. The normalization was performed along each channel separately.

### Network Description

**Figure 1:**
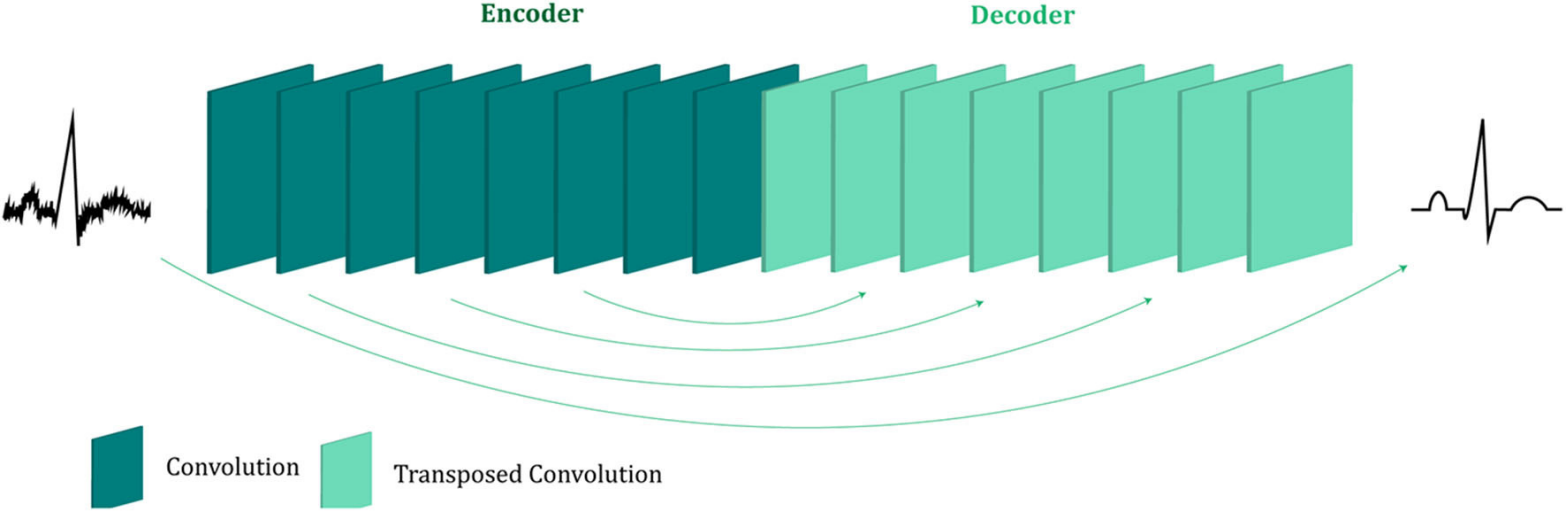
The architecture of the proposed multi-channel fetal ECG denoising network. The network consists of an encoder of eight convolutional layers and a decoder of eight transposed convolutional layers, which are linked symmetrically by skip connections.

The proposed fetal ECG denoising CNN network is illustrated in Figure 1. It consists of an encoder of eight convolutional layers and a decoder of eight symmetric transposed convolutional layers. The network receives a noisy fetal ECG signal as input and delivers a denoised one as output. The convolutional layers act as a feature extractor which captures the abstraction of the fetal ECG while eliminating the noise. Subsequently, the transposed convolutional layers decode the fetal ECG abstraction to recover the signal details. The convolutional layers are symmetrically connected with the transposed convolutional ones via skip connections. The role of the skip connections is two-fold. First, they help back-propagating the gradients to bottom layers, facilitating the training of our deep network. Second, they pass signal content from the bottom to top layers to aid in recovering the signal details.

The non-invasive fetal ECG typically contains a high amount of noise and thus a large denoising patch can lead to more efficient noise removal by using context information from a larger signal region. It was indicated in the literature that the denoising patch is highly correlated with the receptive field of the network, i.e., the region in the input space that a CNN feature can be affected by (11, 32). The receptive field of the network is determined by the kernel size, the depth of the network and whether subsampling or dilation is used in the convolution operations. A common approach to increase the receptive field is to increase the number of layers in the network but this is computationally expensive. We chose to use a relatively deep network of eight convolutional and eight transposed convolutional layers. Since our data are temporal, we adopt one-dimensional convolutions and transposed convolutions. In addition, subsampling by two is performed after each convolutional layer, apart from the first, and upsampling by two after the transposed convolutional layers, apart from the last one. Subsampling operations are not generally preferred in denoising tasks in order to preserve the signal details (10). On the other hand, in our case they lead to a significant increase of the receptive field, necessary for removing the large amount of noise present in the fetal ECG signals. Moreover, in order to exploit the self-similarity of the ECG signals the network should permit the convolutions to extend to several heartbeats. Regarding the kernel size we empirically determined that 15 achieves satisfactory results by being large enough to include sufficient signal information without excessively increasing the number of network parameters. The input and output of the network have dimension 1920 × 4 which corresponds to four-channel ECG of 3.84 s. For non-linearity after each layer, leaky rectified linear units (LeakyRelu) with a slope of 0.2 are utilized. The aforementioned parameter choices led to a receptive field of roughly 4 s that corresponds to 5–10 heartbeats. A detailed description of the network architecture and the parameters is given in Table 1.

**Table 1:** Detailed overview of the proposed network architecture.

	**Layer**	**Output size**	**Filter size**	**Kernel size**
Encoder	Convolution (stride = 1) LeakyRelu(0.2) Convolution (stride = 2) LeakyRelu(0.2) Convolution (stride = 2) LeakyRelu(0.2) Convolution (stride = 2) LeakyRelu(0.2) Convolution (stride = 2) LeakyRelu(0.2) Convolution (stride = 2) LeakyRelu(0.2) Convolution (stride = 2) LeakyRelu(0.2) Convolution (stride = 2) LeakyRelu(0.2)	1920 × 64 1920 × 64 960 × 128 960 × 128 480 × 256 480 × 256 240 × 256 240 × 256 120 × 512 120 × 512 60 × 512 60 × 512 30 × 1024 30 × 1024 15 × 2048 15 × 2048	64 128 256 256 512 512 1024 2048	15 15 15 15 15 15 15 15
Decoder	Transposed Convolution(stride = 2) LeakyRelu(0.2) Transposed Convolution(stride = 2) Addition LeakyRelu(0.2) Transposed Convolution(stride = 2) LeakyRelu(0.2) Transposed Convolution(stride = 2) Addition LeakyRelu(0.2) Transposed Convolution(stride = 2) LeakyRelu(0.2) Transposed Convolution(stride = 2) Addition LeakyRelu(0.2) Transposed Convolution(stride = 2) LeakyRelu(0.2) Transposed Convolution(stride = 1) Addition Linear Activation	30 × 1024 30 × 1024 60 × 512 60 × 512 60 × 512 120 × 512 120 × 512 240 × 256 240 × 256 240 × 256 480 × 256 480 × 256 960 × 128 960 × 128 960 × 128 1920 × 64 1920 × 64 1920 × 4 1920 × 4 1920 × 4	1024 512 512 256 256 128 64 4	15 15 15 15 15 15 15 15

#### Skip Connections

**Figure 2:**
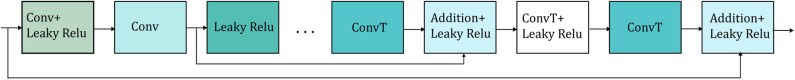
Detailed illustration of the way that the skip connections (represented by the arrows) are applied in the network. Only two skip connections are shown for simplicity. Conv stands for convolution and ConvT for transposed convolution.

In shallow networks transposed convolutions works well for recovering the signal details but as the network goes deeper, they do not longer work satisfactory (9). Our network is deep and heavy subsampling is performed for the sake of increasing the receptive field of the network, resulting in significant loss of signal information. To address this issue, skip connections are added between every two convolutional and mirrored transposed convolutional layers as shown by the arrows in Figure 1. The skip connections carry signal information and account to a great extent for the lost signal details introduced by the subsampling. Moreover, these skip connections allow the gradient update rules to back-propagate to the bottom layers directly, dealing with the gradient vanishing problem occurring in deep architectures. The way that the skip connections are used in the network is depicted in Figure 2.

#### Network Training

For training the network the normalized mean squared error loss was minimized, which is defined as:

(1) $$\begin{array}{r} L=\frac{1}{N^{*}L^{*}M}\overset{N}{\underset{n=1}{\sum }}\overset{L}{\underset{l=1}{\sum }}\overset{M}{\underset{m=1}{\sum }}{\frac{\left({X_{clean}}_{n,l,m}-{X_{denoised}}_{n,l,m}\right)}{{{\bar{X}}_{clean}^{2}}_{n,l}}}^{2}, \end{array}$$

where N is the number of the training data in a batch, L is the number of channels, M is the length of the signals, *X* represents the fetal ECG and ${\bar{X}}^{2}$ is the mean squared amplitude of *X*. In our experiments N = 64, L = 4 and M = 1920. The Adam algorithm was selected (33) as an optimization algorithm while the learning rate was set to 0.00001. The training method that we followed is supervised, meaning that we need clean fetal ECG signals as labels together with the noisy signals. For this reason, the training of the network was performed based only on simulated data. The simulated data were separated in two sets for the training and testing of the method. The training set contains the signals simulated by the modified fecgsyn toolbox based on VCG 1-7 and 449 preprocessed records from 212 subjects of the PTB dataset. The test set contains the simulated signals based on VCG 8-9 from the modified fecgsyn toolbox, plus 100 preprocessed records of 78 subjects of the PTB dataset. The SNR of the training set ranges from −15 to 15 dB. The network was trained for 21 epochs until convergence was reached.

### Performance Evaluation

In the simulated dataset, the performance of the method was evaluated based on the SNR improvement of the fetal ECG signals achieved by the network. The metric is estimated for a channel, *l*, of a signal as:

(2) $$\begin{array}{r} SNR_{imp}=10log_{10}\frac{\overset{M}{\underset{m=1}{\sum }}|{X_{noisy}}_{l,m}-{X_{clean}}_{l,m}{|}^{2}}{\overset{M}{\underset{m=1}{\sum }}|{X_{denoised}}_{l,m}-{X_{clean}}_{l,m}{|}^{2}}. \end{array}$$

The metric was computed for each channel and subsequently averaged over all the ECG channels and test signals.

For real fetal ECG signals there is no ground truth available, because even after the maternal ECG suppression there is still noise present in the signals. Thus, it is impossible to have a gold reference to quantitatively validate the results. Simultaneous scalp recordings may help but they can be performed only during labor. Unfortunately, since our real private dataset was obtained during the second trimester of pregnancy, it was not possible to measure the scalp ECG to have a clean reference. For this dataset, in order to provide some quantitative results along with the qualitative, we decided to generate a surrogate “clean” ground truth signal by calculating the running median of 100 heartbeats. We then measure how well the quality of the denoised signals was enhanced by computing the improvement in SNR performance defined by Equation (2). The metric was calculated for 455 cases, where sufficient QRS complexes were detected for the generation of the “ground truth” signal.

In the Abdominal and Direct Fetal Electrocardiogram Database, since simultaneous scalp measurements are provided together with the non-invasive fetal ECG, the performance of our method was evaluated by comparing with the scalp electrode. The scalp ECG is however a different lead than the abdominal ones and we cannot compare them directly since, even in case of perfect denoising by our method, the morphology of the ECG will not be the same between different leads. Instead, we estimated a denoised scalp ECG as a linear combination of the four abdominal fetal ECG channels:

(3) $$\begin{array}{r} {\hat{X}}_{scalp}=a^{T}X_{denoised},\;a\;={\left(X_{denoised}{X_{denoised}}^{T}\right)}^{-1}X_{denoised}X_{scalp}^{T}, \end{array}$$

where *X*_*scalp*_ is the [1 x 250] scalp ECG and ${\hat{X}}_{scalp}$ the [1 x 250] estimation of the scalp ECG from the abdominal fetal ECG channels. The coefficients of the [4 x 1] linear combination, *a*, were computed on windows of half a second that corresponds to 250 samples. The dimension of *X*_*denoised*_ is 4 x 250. Because the scalp ECG measurements contain considerable amount of noise and this could affect the comparison, we denoised the scalp ECG by high pass filtering followed by averaging of 30 ECG complexes. Nevertheless, we provided comparative results both when the estimation was done based on the noisy scalp ECG as well as on the denoised scalp ECG.

Four different quantitative measures were employed for the comparison, the Pearson correlation coefficient (*R*), the mean squared error (MSE), the mean absolute error (MAE) and the signal-to-noise ratio (SNR). The metrics are defined by the following equations:

(4) $$\begin{array}{rcl} R & = & \frac{cov\left({\hat{X}}_{scalp}X_{scalp}\right)}{\sigma_{{\hat{X}}_{scalp}}\sigma_{X_{scalp}}}, \end{array}$$

(5) $$\begin{array}{rcl} MSE & = & \frac{1}{K}\overset{K}{\underset{i=1}{\sum }}\;{\left(X_{scalp_{i}}-{\hat{X}}_{scalp_{i}}\right)}^{2}, \end{array}$$

(6) $$\begin{array}{rcl} MAE & = & \frac{1}{K}\overset{K}{\underset{i=1}{\sum }}|X_{scalp_{i}}-{\hat{X}}_{scalp_{i}}|, \end{array}$$

(7) $$\begin{array}{rcl} SNR & = & \;10log_{10}\frac{\overset{K}{\underset{i=1}{\sum }}|{X_{scalp}}_{i}{|}^{2}}{\overset{K}{\underset{i=1}{\sum }}|{X_{scalp}}_{i}-{\hat{X}}_{scalp_{i}}{|}^{2}}, \end{array}$$

where *cov* stands for the covariance, σ the standard deviation and K the length of the signals. The metrics were computed for the five signals of the database and subsequently averaged to obtain one final value.

### Reference Methods

Our method was evaluated in comparison with 3 other ECG denoising methods. The first method is the single-channel CNN denoising network, where each fetal ECG channel is denoised separately (21). The second method is a wavelet denoising algorithm that removes the noise by thresholding the detail coefficients after the signal decomposition. The symlet wavelet was selected due to its resemblance with an ECG, while a fixed threshold was used, estimated by the minimax principle (34). The last method is the widely used beat-to-beat averaging method. We selected to average 30 beats similar to the averaging performed by the STAN method (35). The QRS complexes were detected by a Pan Tompkins detector in the clean fetal ECG signals and not the noisy ones because we do not intend to assess the performance of the QRS detector but the performance of the averaging method. However, we should note that it is not guaranteed that the QRS complexes can be accurately estimated in the presence of acute noise.

## Results

### Performance on Simulated Signals

**Figure 3:**
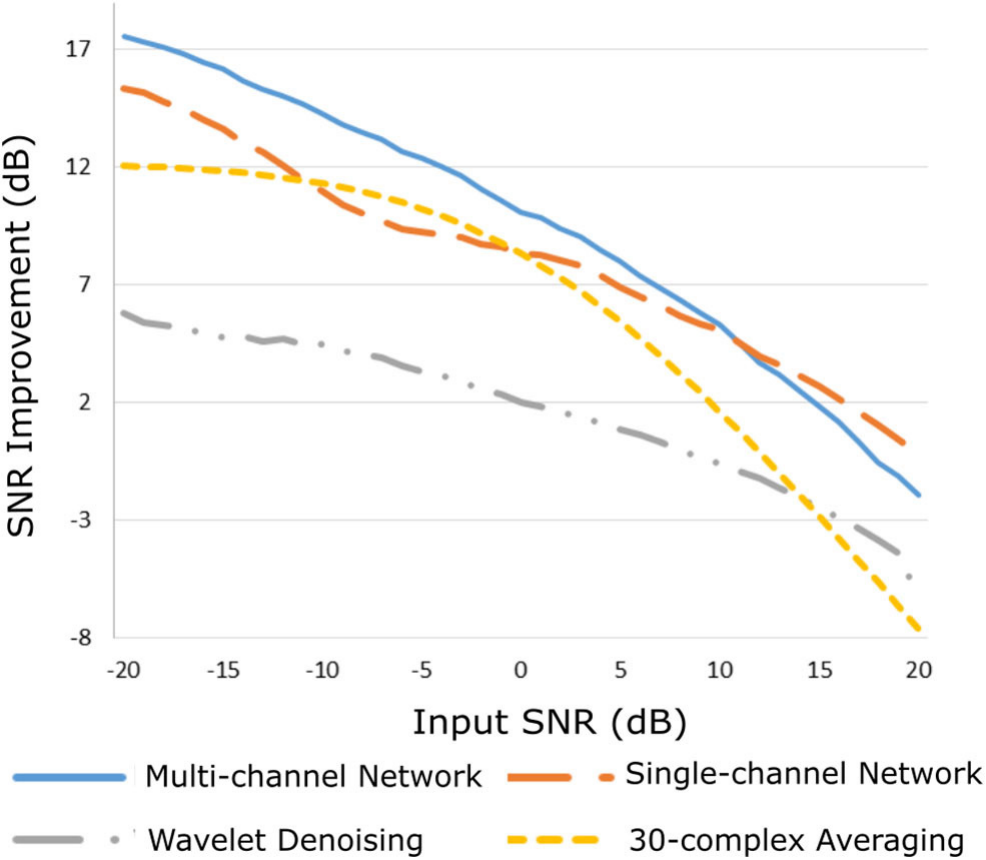
Performance of the proposed multi-channel convolutional network in comparison with other denoising methods in terms of improvement in SNR of the denoised fetal ECG signals when compared with the noisy ones.

The improvement in SNR performance of the proposed network in comparison to the other denoising algorithms, for input SNR from −20 to 20 dB, is illustrated in Figure 3. As demonstrated in this figure, the CNN network provides a considerable amount of SNR improvement throughout the whole range of input SNR. The proposed method outperforms the beat-to-beat averaging and the wavelet denoising methods for all the input SNR values. This was anticipated because the averaging method does not preserve individual variations among complexes, while our method is capable of doing so. Moreover, the wavelet denoising distorts the signal amplitude, whereas the proposed network preserves it better. The multi-channel network additionally outperforms the single-channel nearly for the whole range of input SNR values. More specifically, for input SNR <0 dB the multichannel algorithm provides an SNR improvement of at least 10 dB with respect to the input signal and at least 2 dB further improvement as compared to the single-channel method. As the input SNR increases the performances of the two methods become gradually comparable, while for input SNR more than 11 dB the single-channel network slightly surpasses the multi-channel. This was something to expect because for signals of lower quality, information from multiple channels will be beneficial for recovering the ECG structure. On the other hand, if a fetal ECG channel has sufficiently high quality not only the other channels are unnecessary for denoising it but could also slightly affect the quality of the denoised fetal ECG, especially in case their SNR is low. This can be explained better by the following: By using any set of three linearly independent ECG leads, the VCG can be constructed, which is the three-dimensional representation of the electrical activity of the heart. A VCG can explain about roughly 90% of an ECG signal (36). This means that when a signal is reconstructed from different channels, 10% of the signal information should be considered as not reconstructable. In case of very high signal quality, the single-channel denoiser can perform better than the multi-channel since it could theoretically reconstruct 100% of the signal.

By observing Figure 3, we see that, for all methods, there is an input SNR for which the denoisers decrease the SNR. This input SNR value is 9, 12, 18, and 20 dB for the wavelet, averaging, multi-channel network and single-channel network denoising methods, respectively. Since it is not common to obtain fetal ECG signals of very high quality (more than 18 dB), we do not consider it as a limitation of our method. We additionally noticed that there is a upgoing trend for the SNR improvement metric as the input SNR decreases. However, we did not test for signals of quality even lower than −20 dB because real fetal ECG signals typically do not have quality less than −20 dB.

**Figure 4:**
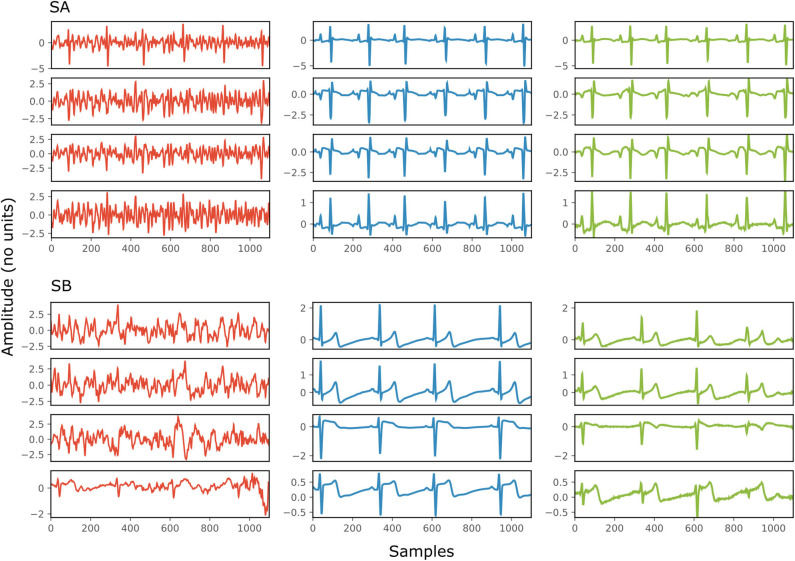
Denoising results by the proposed method for two simulated signals (SA and SB) of the test dataset. For both signals: each panel in the left presents one channel of the noisy four-channel fetal ECG signal (red), in the middle the corresponding channels of the clean signal (blue) are shown and in the right the denoised fetal ECG signal by our network (green). The horizontal axis depicts the samples at 500 Hz, while the vertical the amplitude of the signals. The SNR values of the noisy and the denoised fetal ECG for both signals are given in Table 2 (SNRin and SNRout, respectively).

Figure 4 depicts two typical denoising results from our test dataset. The SNR values of the signals before and after denoising are provided in Table 2. Note that in Figure 4 the vertical axes limits for the noisy signals differ from those of the ground truth and denoised signals for better visualization. However, the axes limits for the clean and denoised fetal ECG are the same to allow for their comparison. As can be noted, the network suppresses the noise in a great extent for both signals simulated-A (SA) and simulated-B (SB). In the case of signal SA the similarity of the network's output with the clean signals is very high for all channels and all ECG waves are clearly distinguishable. Even for channel 4, with input SNR of −12 dB, the network provides a high-quality result, since it combines all channels to reconstruct it. For signal SB the majority of ECG channels have very low quality (around −9 dB). The SNR after denoising with our network is significantly higher (3.75 dB on average). However, we notice some distortion on the signal amplitude, while particularly the P-waves are suppressed by the network. Moreover, despite channel 4 having the least amount of noise before entering the network, we observe the least improvement after denoising, evidencing that indeed the network's output is obtained through combination of information from all leads.

**Table 2:** The SNR values in dB for the four channels of the simulated signals depicted in Figure 4, before (SNRin) and after (SNRout) denoising.

**Channel**	**Signal SA**		**Signal SB**	
	**SNRin**	**SNRout**	**SNRin**	**SNRout**
1	1	17	−9	5
2	−2	13	−9	4
3	−3	14	−10	3
4	−12	8	0	3

### Evaluation on Real Fetal ECG Signals

**Figure 5:**
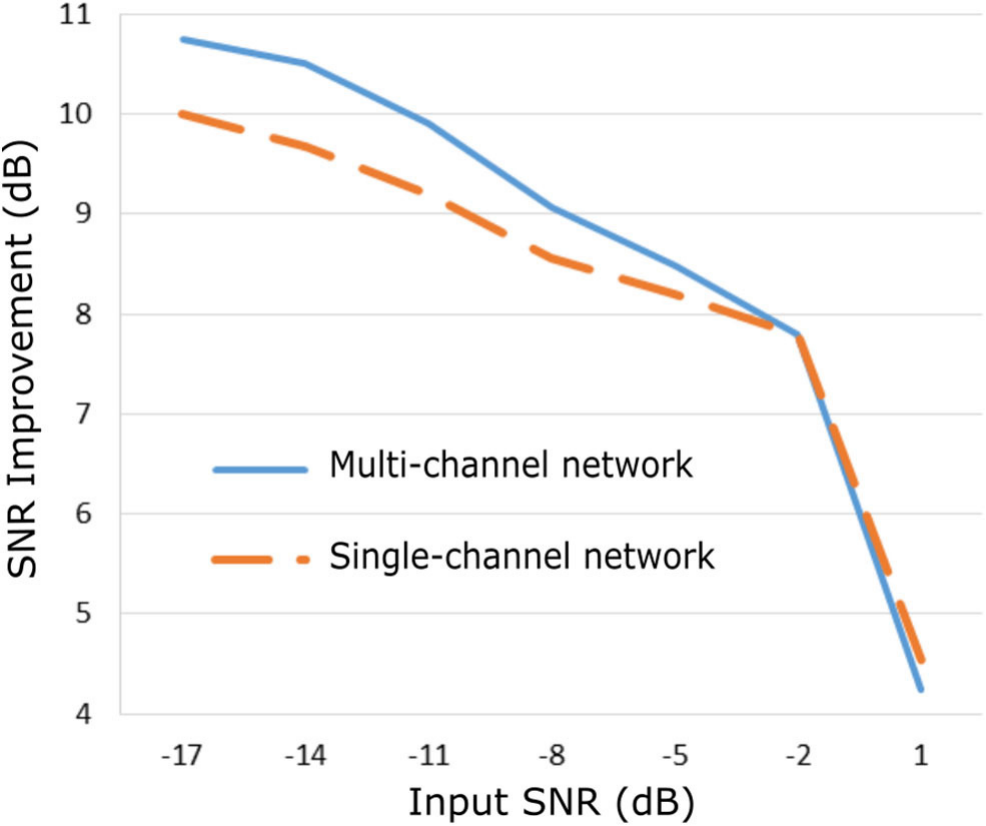
Performance of the proposed fetal ECG denoising method in a large real dataset (28) in terms of improvement in SNR of the denoised signals.

The proposed method was evaluated on our extensive non-invasive fetal ECG dataset (28) and the results are presented in Figure 5. Figure 5 illustrates the improvement in SNR for input SNR ranging from−17 to 1 dB. The input SNR corresponds to the SNR of the noisy fetal ECG signals when we assume that the ground truth signal is the running median of 100 heartbeats. We need to stress that this is not the actual SNR of the signals but merely an approximation of it. In fact, the more noise is present in the signals or the more physiological variation, the less accurate the constructed “clean” signal is. Examining the Figures 3, 5, where the performance in the simulated dataset is illustrated, we observe an analogy between them. In both graphs the multi-channel denoiser surpasses the single-channel for lower input SNR while for higher SNR values the two methods perform comparably. The performance improvement as compared to the single-channel approach is lower for the real signals than for the simulated ones but this might be due to the lack of actual ground truth signals for comparison. By all means the evaluation in this dataset is suboptimal but it provides a performance indicator in a large real dataset.

**Figure 6:**
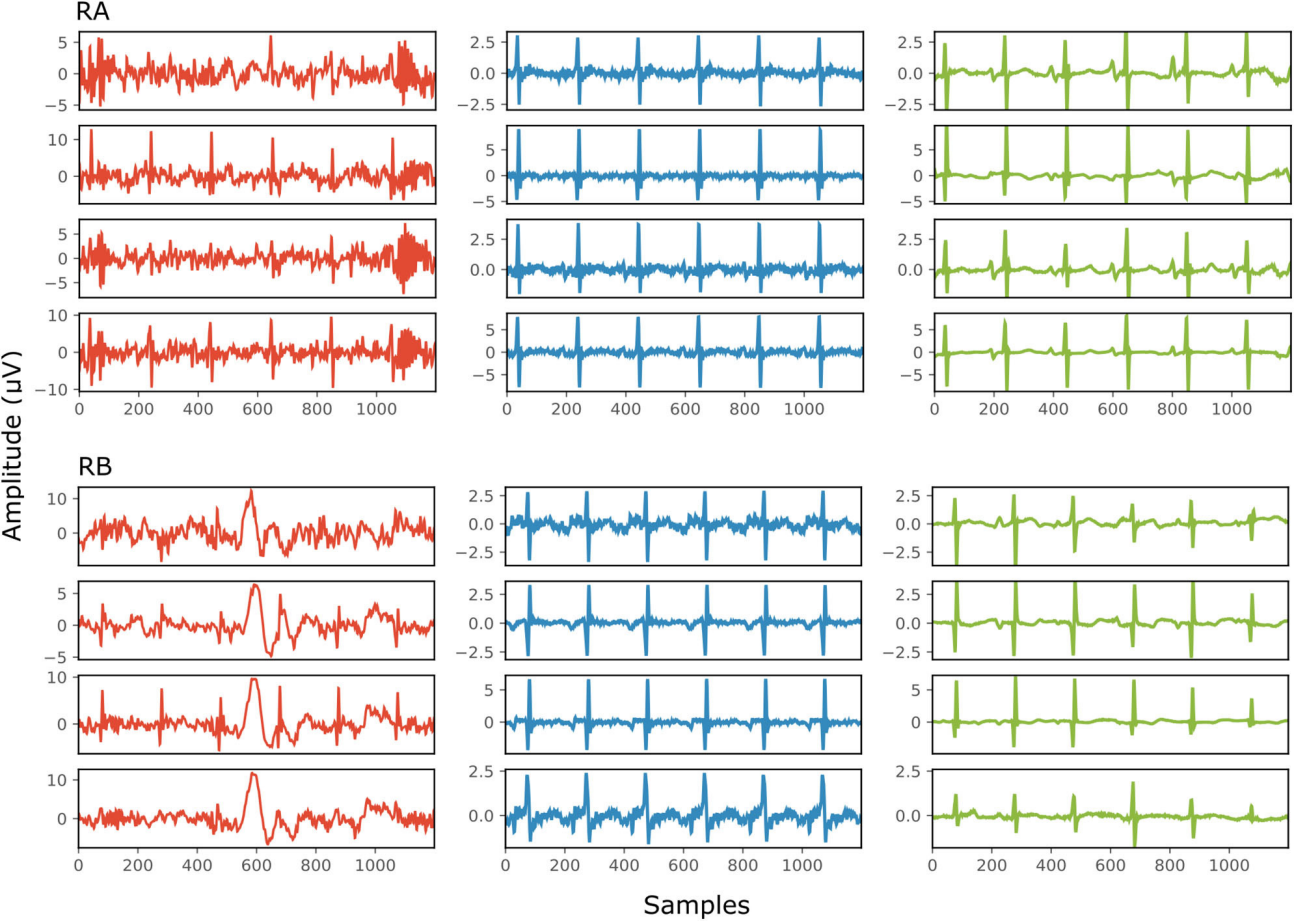
The results of denoising two signals (RA and RB) from our private real fetal ECG dataset (28). For each signal: the noisy four-channel fetal ECG signal extracted from the abdominal measurements is presented in the left (red), the running median of 100 heartbeats for each channel in the middle (blue) and the denoised fetal ECG signal by our network in the right (green). The horizontal axis depicts the samples at 500 Hz, while the vertical the amplitude of the signals in μV. The SNR values of the noisy fetal ECG signals (SNRin) together with the values for the denoised ones (SNRout) are given in Table 3.

Figure 6 demonstrates the result of denoising two signals of this database, while Table 3 provides the corresponding SNR values before and after denoising. Note that the vertical axes limits for the noisy signals differ from the ones of the “clean” and denoised ones for clearer visualization. Both signals in Figure 6, especially signal real-B (RB), have a significant amount of noise before denoising (see Table 3). The “clean” reference signals as well contain few noise but in most of them the ECG morphology is relatively clear. On the other hand all the possible variations among the successive complexes is lost due to the heavy averaging performed. The multi-channel network achieved a fairly remarkable result in denoising those signals. Comparing the morphology of the denoised with the “clean” reference signals, the various ECG waves and segments correspond relatively well. In this comparison, we acknowledge that the running median of 100 heartbeats is not the gold standard. However, the averaging of the heartbeats brings evidence for the location of the ECG waves, especially the P-waves, information that cannot be seen in the noisy signals. It is important to recognize that in the denoised signals by our network, these locations seem to correspond with the locations in the median signals. As a matter of fact, the denoised signals appear to exhibit better quality and clearer morphology than the reference. Some morphological features seem to be distorted, as we can see in Figure 6 for signal RB. However, the overall performance in those low-quality signals is relatively good.

**Table 3:** The SNR values in dB for the four channels of the real signals depicted in Figure 6, before (SNRin) and after (SNRout) denoising.

**Channel**	**Signal RA**		**Signal RB**	
	**SNRin**	**SNRout**	**SNRin**	**SNRout**
1	−8	3	−13	3
2	−3	3	−8	5
3	−8	2	−6	6
4	−1	6	−14	1

**Figure 7:**
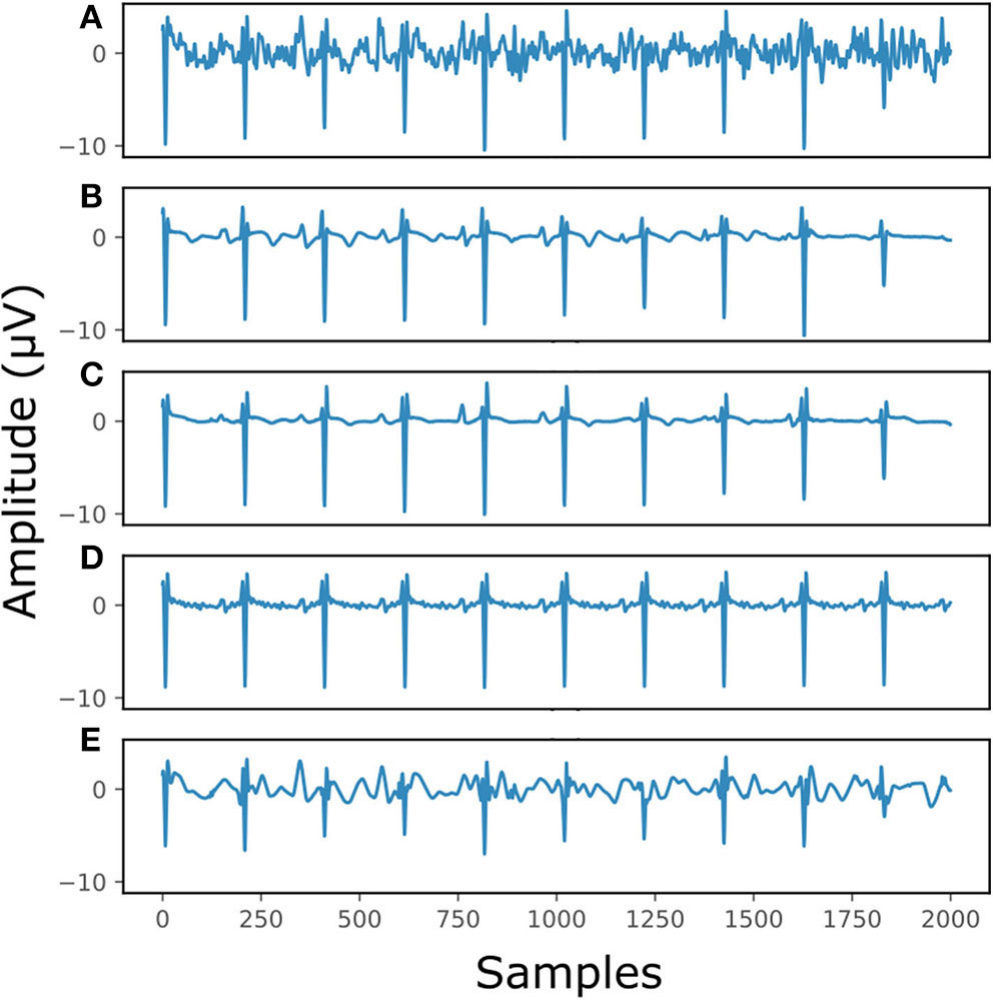
The result of denoising a real fetal ECG signal from our private dataset (28) with different denoising algorithms. For simplicity, only one channel is displayed. The panels show: **(A)** the noisy extracted fetal ECG, **(B)** the denoised signal by the proposed method, **(C)** the denoised signal by the single-channel denoising network, **(D)** the result of 30-complex averaging and **(E)** the result after wavelet denoising. The horizontal axis depicts the samples at 500 Hz, while the vertical the amplitude of the signals in μV.

Figure 7 illustrates the performance for a fetal ECG signal of on non-invasive fetal ECG dataset in comparison to the single-channel network, 30-complex averaging and wavelet denoising. For simplicity we present only one channel out of the four. As shown in the figure, all methods provide a noise-free result. However, our method retains the individual ECG complex differences as opposed to the averaging method and does not distort the signal amplitude as opposed to wavelet denoising. In addition, the morphology of the denoised ECG is clearer in our case. The single-channel network provided a similar result to the multi-channel for this signal.

The performance of the network on the Abdominal and Direct Fetal ECG Database is illustrated in Table 4. The scalp ECG was compared with the aforementioned linear combination of abdominal signals, as described in Equation (3). In Table 4 we provide the results of this comparison for 2 cases; when we used the original scalp ECG and when we denoised it. For each performance metric the values before and after denoising with the multi-channel and single-channel network are presented, while with bold the best performing method is marked.

**Table 4:** Performance of the multi-channel CNN network vs. the single-channel one on the Abdominal and Direct Fetal ECG Database in terms of comparison of the scalp ECG with a scalp estimated from the denoised abdominal fetal ECG.

**Metric**	**Original scalp ECG**			**Denoised scalp ECG**		
	**Noisy input**	**Multi-channel output**	**Single-channel output**	**Noisy input**	**Multi-channel output**	**Single-channel output**
R	0.53	**0.66**	0.65	0.74	**0.87**	0.85
MSE (μV^2^)	555.8	**440.7**	449.3	116	**62.1**	68.4
MAE (μV)	15	**12.9**	13	7.3	**5.4**	5.5
SNR (dB)	1.5	**2.7**	2.5	3.7	**6.4**	6.1

*The best performing method is marked with bold*.

First, we believe that denoising of the scalp ECG was important to allow for better comparison with the scalp ECG estimation from the denoised abdominal leads. By averaging 30 successive ECG complexes we might have lost some morphological variations among the successive beats of the scalp lead but achieved significant quality improvement. Even the scalp ECG approximated by the noisy fetal ECG signals has better resemblance with the denoised scalp lead, e.g., correlation coefficients of 0.74 vs. 0.53. Second, we observe that both the multi-channel and single-channel networks achieve significant quality improvement of the fetal ECG signals for all the metrics presented in Table 4. We should note here once more that by no means the scalp estimation is expected to be the same with the scalp ECG even after perfect denoising, because the latter is a different lead than the abdominal leads. Last, the multi-channel network outperforms the single-channel in terms of all computed performance metrics. However, the differences are relatively small. It might be because the extracted fetal ECG signals already have decent quality and, as we have already found in simulated signals, employing multiple channels is more advantageous in cases of signals exhibiting lower SNR. Larger difference was found regarding the MSE metric (62.1 vs. 68.4 μV^2^), indicating that the single-channel network may provide more outliers, while the multi-channel a smoother outcome.

**Figure 8:**
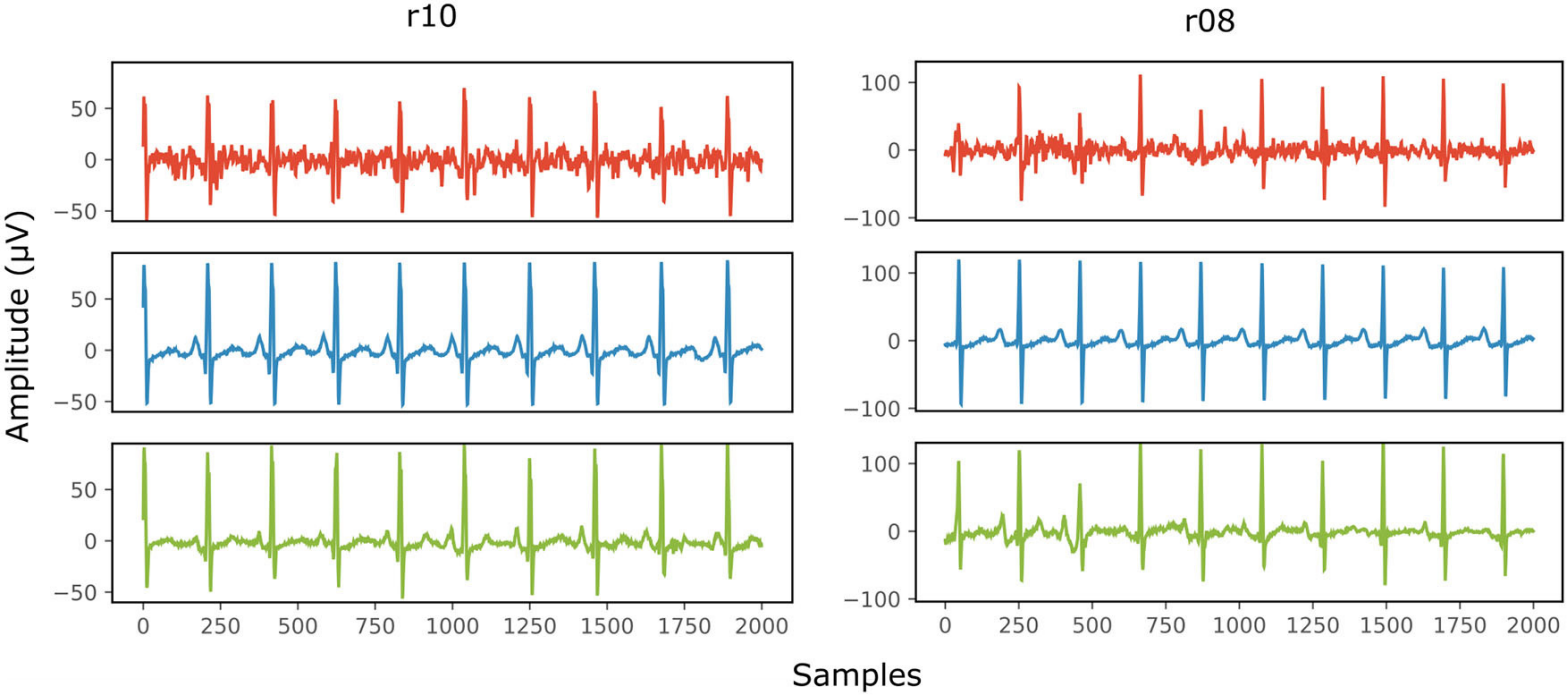
Comparison of the scalp ECG with an estimation of it as a linear combination of the abdominal fetal ECG for 2 records (r10 and r08) of the Abdominal and Direct Fetal ECG Database. In the first row (red) the scalp ECG estimated from the noisy fetal ECG is presented, in the second (blue) the ECG as measured by the scalp electrode (running average of 30 complexes) and in the last one (green) the scalp ECG estimated from the denoised fetal ECG by the proposed network. The horizontal axis depicts the samples at 500 Hz, while the vertical the amplitude of the signals in μV.

Figure 8 provides two qualitative results of the scalp estimation, when fetal ECG denoising is performed with the proposed multi-channel method. In both cases, the scalp estimated by the denoised fetal ECG is free from noise and the individual waves and intervals correspond relatively well to those of the scalp ECG. We do not expect absolute correspondence, not only because the scalp ECG is a different lead, but also because it was averaged over 30 complexes.

## Discussion

We have proposed a CNN network for postprocessing non-invasively extracted multi-channel fetal ECG signals to improve their quality. The non-invasive fetal ECG is substantially contaminated by various noises, even after the application of various signal processing tools proposed in literature, such as maternal ECG suppression. The low quality of the fetal ECG is the principal reason that the applicability of non-invasive fetal electrocardiography in clinical practice is limited. The suggested multi-channel network was trained on a wide dataset of simulated four-channel ECG signals, with SNR ranging from−15 to 15 dB, while it was extensively validated both on simulated as well as on real datasets.

Experiments on simulated data showed a significant improvement in the quality of the noise-corrupted fetal ECG signals. The network combined information from all the channels to efficiently remove the noise and uncover the ECG signal morphology even in the presence of acute noise. However, the network suppressed some morphological characteristics in cases there was not sufficient content for denoising i.e., when most signal channels were severely corrupted. The multi-channel network outperformed the single-channel (21) in cases of low SNR of the input signals, while for SNR more than 11 dB the single-channel network exhibited slightly better performance. This behavior could be anticipated. A multi-lead signal configuration captures the spatiotemporal nature of the cardiac electrical activity. For low quality signals this is beneficial as more signal information can be exploited to better reconstruct each channel. However, if we wish to denoise a channel that already has high quality, using spatiotemporal content may be not always the best choice. Nevertheless, it is very uncommon in practice to obtain fetal ECG of such high quality. Yet, in case this would happen, the output of the multi-channel network would still be of such quality that it could be used for further clinical interpretations.

The evaluation of our network on a large real fetal ECG dataset showed an analogous behavior to that on the simulated data; for low quality fetal ECG the multi-channel network outperformed the single-channel, while for higher SNR the performances of the networks were comparable. We cannot make a direct comparison because the evaluation method for the real signals was suboptimal. We are aware that the approximation of the ground truth signals with the running average of 100 heartbeats was not very accurate. However, it gave us an indication that the method is efficient in real data too. We additionally presented some qualitative denoising results for two signals of this database in Figure 6 to support our claim. The network outputted clean denoised signals with good correspondence of the individual ECG waves between the reference and denoised signals. A few recordings in our dataset had input SNR that was even lower than−17 dB. Based on visual analysis of the output of our proposed denoiser, we could argue that the performance of the denoiser breaks down at these very low signal quality levels and the network is no longer capable of reconstructing a reliable fetal ECG. This limitation probably comes from the fact that the network was trained for input SNR range of−15 to 15 dB. Thus, the network did not learn to remove efficiently the noise when the quality of the signals is even lower. This indicates that we might need to perform experiments for a wider SNR input range. However, the capacity of the network might no longer be sufficient for handling such an ample range of signal qualities and further research is needed to evaluate this.

The CNN network was additionally evaluated in the Abdominal and Direct Fetal ECG Database. Simultaneously recorded scalp ECGs were compared to an estimated scalp ECG from the denoised abdominal channels, also here demonstrating that the method can provide significant quality improvement of the noisy fetal ECG signals. Comparison of the performances of the multi-channel and single-channel networks for this database, revealed that they achieve comparable results, probably because the input signals were of relatively good quality. It is difficult to compare the performances between the two real datasets for several reasons. Most importantly, the sizes of the two datasets differ a lot (455 vs. 5) and so do the gestational ages of the subjects (18-24 vs. 38-41 weeks).

As mentioned in the introduction and also in (21), the shortcoming of denoising single-channel fetal ECG with a convolutional network is that the network can output signals that look as if they were ideally denoised, but that can have “fake” waves that can differ both in location and polarity when compared to the actual ECG waves. This happens mostly when the quality of the input signals is relatively low and the network, not having enough signal information, reconstructs a clean signal from unreliable information in the encoded latent space. We demonstrated that by employing multichannel signals this problem is eliminated to a large extent. When the quality of the signals is very low, the amplitude of the small signal waves, like the P-wave and T-wave, and less often of the R-peaks in the denoised signals can be distorted rather than “fake.” This means that some waves may be virtually absent, or the output does not even resemble an ECG anymore. This makes the method safer to use in clinical practice, because clinicians will typically discard a distorted signal but a signal that looks like a high-quality ECG but in fact contains “fake” information might lead to erroneous decision-making.

To summarize, we have shown the potential of deep CNNs for removing noise from non-invasive multi-lead fetal ECG. We validated the method on a wide dataset of simulated but also real recordings with both early as well as late gestational ages (18 to 24 and 38 to 41 weeks). Primarily, we demonstrated that employing multi-channel information for denoising does not only lead to more clean signals but also to more reliable results, when compared to single-channel information. The main advantage of the method is that, as opposed to the widely used averaging method, no prior processing of the signal is needed to extract the locations of the R-peaks and variations in ECG morphology among consecutive heartbeats are preserved. This is especially important in case that arrhythmias are present. Up to now, arrhythmia is assessed through echocardiography because the averaging that was performed to enhance the quality of the fetal ECG hinders its application for arrhythmia analysis. Moreover, the quality of the denoised signals is high enough to allow for measuring the timing of intervals, like the PR and QT interval. However, in order to confirm this, we need to perform a thorough comparison of the ECG intervals between the denoised and the clean signals. If we wish to obtain reliable results, a large annotated dataset is necessary, but this requires time and experts to perform these annotations.

Certainly, there is room for improvement of our method. Most importantly, the capacity of the network could be further increased to handle even more noisy signals. Moreover, we can explore denoising directly the raw abdominal signals, without cancelling the maternal ECG. Most probably a more complex network architecture is needed for such a task and appropriate data for training.

## Conclusion

An end-to-end trained deep CNN network was presented for denoising of fetal ECG signals. Convolutions and transposed convolutions were combined in the network, modeling the denoising problem as an encoding of primary signal content and subsequent decoding to recover details. Essentially, we proposed to employ spatiotemporal information in the ECG signal by using multiple ECG leads simultaneously as input to the network. The network then learned how to combine the input channels and deliver a reliable clean ECG as output. Experiments on simulated as well as in real data showed that the network can achieve a substantial quality improvement of the noisy signals and outperform a single-channel alternative.

## Data Availability Statement

Data are available from the Data Governance Board of the Máxima Medical Centre to researchers who can demonstrate that they are qualified to use confidential data. Requests to access the data should be addressed to the corresponding author or the Data Governance Board at the Máxima Medical Centre (J. Luime, j.luime@mmc.nl). Other data used in the study can be found in: https://archive.physionet.org/physiobank/database/adfecgdb/?C=S;O=A.

## Ethics Statement

The studies involving human participants were reviewed and approved by The study was approved by the Máxima Medical Centre institutional review board (NL48535.015.14). Participants were included in the study after written informed consent had been obtained. The patients/participants provided their written informed consent to participate in this study.

## Author Contributions

EF is a Ph.D. student and has done the work of the paper, while RV closely supervised the work while giving ideas, feedback, and guidance.

## Conflict of Interest

RV has shares in Nemo Healthcare BV, The Netherlands. The remaining author declares that the research was conducted in the absence of any commercial or financial relationships that could be construed as a potential conflict of interest.
